# Woodhouse-Sakati Syndrome Presenting With Psychotic Features After Starting Trihexyphenidyl: A Case Report

**DOI:** 10.7759/cureus.27576

**Published:** 2022-08-01

**Authors:** Mohammed A Aljaffer, Ahmad H Almadani, Mohammad AlMutlaq, Abdulaziz Alhammad, Ahmed S Alyahya

**Affiliations:** 1 Department of Psychiatry, College of Medicine, King Saud University, Riyadh, SAU; 2 College of Medicine, King Saud University, Riyadh, SAU; 3 Department of Radiology, King Faisal Specialist Hospital and Research Center, Riyadh, SAU; 4 Department of Psychiatry, Eradah Complex for Mental Health, Riyadh, SAU

**Keywords:** dystonia, case report, drug-induced psychosis, trihexyphenidyl, woodhouse-sakati syndrome

## Abstract

Woodhouse-Sakati syndrome is a rare, autosomal recessive, multisystemic disorder first identified as a constellation of hypogonadism, mental retardation, diabetes, alopecia, deafness, and electrocardiogram abnormalities.

We report a case of a 33-year-old woman who was born to consanguineous parents. She is suffering from hypergonadotropic hypogonadism, extrapyramidal symptoms, hypothyroidism, alopecia, and sensorineural hearing loss. Her MRI showed iron depositions in globus pallidus bilaterally. She underwent genetic testing and was diagnosed with Woodhouse-Sakati syndrome. She was started on trihexyphenidyl to treat her extrapyramidal symptoms. A few months later, she started to have psychotic symptoms in the form of auditory hallucinations and delusions of persecution.

Although she exhibited psychotic symptoms after starting trihexyphenidyl, it is less likely to be causing her symptoms since the symptoms started a few months after taking the medication and she was not on high doses. Thus, it is more likely to be a part of Woodhouse-Sakati syndrome.

## Introduction

Woodhouse-Sakati syndrome (WSS) is a rare, autosomal recessive, multisystemic disorder. It was first identified in 1983 by Woodhouse and Sakati in six patients of a highly inbred family [1]. The syndrome has a constellation of multiple characteristics, including endocrinological, ectodermal, and neurological findings [2]. The endocrinological dysfunction includes hypogonadism, decreased insulin-like growth factor 1 (IGF-1), diabetes mellitus, and hypothyroidism [2,3]. The ectodermal manifestations include alopecia and anodontia, while the neurological symptoms include chorea, dysarthria, sensorineural hearing loss, intellectual disability, and dysphagia [3,4]. As time went by, many cases reported similar descriptions. However, some patients exhibited variations in the age at disease onset, manifestations, and symptoms severity, even among the affected members of the same family. Such variations may result in a delay of the diagnosis or sometimes misdiagnosis. The syndrome is caused by a mutation in the gene DCAF17, which encodes DDB1- and CUL4-associated factor 17, previously known as c2orf37, on chromosome 2q31.1 [5,6,7]. The latest global prevalence of WSS was approximately 88 reported patients, including more than 40 affected families; fifty-one had a molecular confirmation of the diagnosis [3]. Most of the cases were reported from Saudi Arabia [2,4,5,8]. However, other cases from Asia, the Middle East, and Europe were reported as well: Qatar [9,10], Portugal [11], Tunisia [12], Pakistan [13], India [14], Italy [15] and Turkey [16]. 

Trihexyphenidyl is an antimuscarinic medication that is often used to manage extrapyramidal symptoms in Parkinson’s disease. Like any other medication, this medication has many side effects and is considered a medication that can be abused [17]. Different patients show different responses to this medication regarding side effects; some exhibit negative effects such as hallucinations and delirium, others show positive effects such as euphoria, while the rest do not experience any side effects. [17]

This paper aims to present an unusual case of a patient with WSS who presented with psychotic symptoms after being treated with trihexyphenidyl.

## Case presentation

This is a 33-year-old woman born to consanguineous parents (second-degree relatives). She exhibited dysmorphic features, including a long triangular face, low-set ears, sparse eyebrows, widely spaced eyes, and teeth crowding. She surpassed the age of puberty without developing secondary sexual characteristics and primary amenorrhea. Subsequently, she was started on hormonal therapy. Moreover, since early childhood, she has suffered from hearing difficulties. Although she had normal neurodevelopmental growth, her school performance was described to be “less than average,” as per her mother. She enrolled in a public university but then was asked to drop out and since then has stayed home.

In 2017, in addition to the gynecology service, she started following up with multiple services at our center, including dermatology, neurology, speech and language team, and audiology. Upon examination, she had dystonic gait, dysarthria, and oromandibular and cervical neck dystonia. She also exhibited bilateral ptosis and bilateral leg swelling. Her laboratory tests (Table 1) showed high thyroid stimulating hormone (TSH) and low free thyroxine 4 (FT4), indicating hypothyroidism. The HbA1C (glycated hemoglobin) was 6%, indicating a pre-diabetic state. Other results showed high follicle-stimulating hormone (FSH) and normal luteinizing hormone (LH), suggesting hypergonadotropic hypogonadism.

**Table 1:** Laboratory findings ^above reference range; *below reference range; TSH: thyroid stimulating hormone; FT4: free thyroxine 4; FSH: follicle-stimulating hormone; LH: luteinizing hormone; HgbA1c: hemoglobin A1c

Labs	Level	Reference range	Date obtained
TSH	7.3 mIU/L^	0.25–5.0 mIU/L	3-6-2021
FT4	4.8 pmol/L*	11.4–22.7 pmol/L	3-6-2021
FSH	38.8 IU/L^	1.5 to 12.4 IU/L	22-8-2021
LH	15.25 IU/L	5-25 IU/L	22-8-2021
HgbA1c	6%*	4–5.6%	22-8-2021

**Figure 1:**
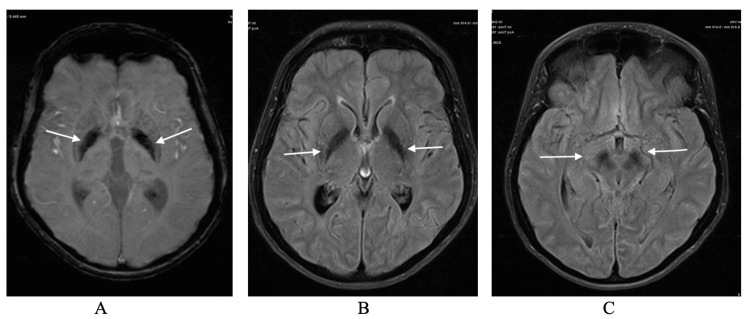
Image A is showing blooming artifact on Susceptibility Weighted Images (SWI) representing iron depositions involving the globus pallidus bilaterally. Images B and C are demonstrating low signal intensity on T2-weighted images involving the globus pallidus bilaterally

During the work-up of sensory-neural hearing loss, an MRI scan of the brain showed increased iron depositions in globus pallidus bilaterally, suggesting a metabolic neurodegenerative disease (Figure 1). Upon referral for genetic assessment and analysis in 2018 using Whole Sequencing Examination (WES), she was found to have a homozygous variant c.436del p. (Ala147 Hists*9) in the DCAF17 gene and was diagnosed with WSS. Afterward, her sister, who is also following in our center for primary amenorrhea, underwent WES and was found to have the same mutation. In the following year, and during neurology follow-up, she complained of dysarthria and eating difficulties with lower facial spastic movements. By October 2019, she was prescribed trihexyphenidyl 2 mg twice daily. Two weeks later, the patient’s family started to notice that she had an irritable mood and diminished activity, which was never noted before. The family then decided to stop the medication without any medical advice. 

About a year later, as her extrapyramidal symptoms and low mood persisted, she visited the neurology and neuro-cognitive clinics for follow-up. In addition to escitalopram 10 mg daily, trihexyphenidyl was prescribed again with gradual doses reaching up to 2 mg twice daily. In March 2021, four months after re-taking trihexyphenidyl, she was seen by a psychiatrist, and the history was obtained from her, and her mother (her legal custodian) since the patient had dysarthria and was difficult to be understood, which made it hard to do comprehensive cognitive assessment due to language barrier. However, she was oriented to time, place, and person. During the interview, she acknowledged that she had been having auditory hallucinations. The history also revealed delusions of persecution, believing that her family members were always talking about her, and that her older sister was plotting against her. She was also convinced that a group of people was going to kidnap her. They denied any other symptoms such as restlessness or agitation, and the assessment concluded that the patient did not have any typical picture of delirium. Moreover, the patient was not helping around the house as she used to, in addition to poor self-hygiene. Thus, she was given olanzapine 5 mg once daily. In the next follow-up visit, she and her mother reported improvement in symptoms, and olanzapine was increased to 10 mg daily. Her psychiatric symptoms have improved significantly since then.

## Discussion

The patient exhibited the classic symptoms of WSS, manifesting with neurological, endocrinological, and ectodermal symptoms as well as laboratory findings [2,3,10,12]. Although it was previously reported that most patients with WSS would present with diabetes mellitus by late teens or early adulthood, the patient only had laboratory findings suggestive of pre-diabetes [3,6,8,9]. Similar imaging features of iron depositions in the globus pallidus were also seen on brain MRI [18,19]. The patient also exhibited psychotic features. Only two cases in the literature were found to have psychotic features [2,15]. In both cases, the reason for developing psychosis was not explored thoroughly. 

Trihexyphenidyl, the medication that our patient is using, is an anticholinergic medication often used as the first line to control extrapyramidal symptoms in psychotic patients [3,20]. The patient’s sister, who was found to have the same condition, did not experience any extrapyramidal or psychotic symptoms.

Moreover, our patient’s psychotic features were observed after starting trihexyphenidyl for a few months. All the previously mentioned points raise the likelihood that trihexyphenidyl may be the trigger of psychosis. In the literature, it has been reported that higher doses of this medication could induce psychotic features [20,21]. Although trihexyphenidyl cannot be excluded as the cause of her psychotic symptoms, it is a less probable one since the symptoms started after months of using the medication and she was not taking high doses of it. Nevertheless, the patient’s mother - who was taking care of her at the time - reported compliance with the prescribed dose of trihexyphenidyl. It has also been suggested that psychiatric features should be added to the spectrum of WSS [15]. Further studies and exploration of psychiatric features in such patients would help in preventing the delay of diagnosis and possible misdiagnosis. 

## Conclusions

This is the second case of WSS in the current literature who develops psychotic symptoms. Although her symptoms developed after starting trihexyphenidyl for a few months and she was not taking high doses of the medication, trihexyphenidyl cannot be totally excluded as the cause of her symptoms. Based on our findings, we suggest that patients with WSS should be observed closely for any emerging psychotic features, as they may be a part of the syndrome.
